# Menopause leads to elevated expression of macrophage-associated genes in the aging frontal cortex: rat and human studies identify strikingly similar changes

**DOI:** 10.1186/1742-2094-9-264

**Published:** 2012-12-03

**Authors:** Miklós Sárvári, Erik Hrabovszky, Imre Kalló, Norbert Solymosi, István Likó, Nicole Berchtold, Carl Cotman, Zsolt Liposits

**Affiliations:** 1 Laboratory of Endocrine Neurobiology, Institute of Experimental Medicine, Hungarian Academy of Sciences, Szigony utca 43, Budapest, 1083, Hungary; 2 Faculty of Information Technology, Pázmány Péter Catholic University, Práter utca 50/A, Budapest, 1083, Hungary; 3 Faculty of Veterinary Science, Szent István University, István utca 2, Budapest, 1078, Hungary; 4 Pharmacology and Drug Safety Research, Gedeon Richter Plc, Gyömrői út 19-21, Budapest, 1103, Hungary; 5 Institute for Brain Aging and Dementia, University of California, Irvine, CA, 92697-4540, USA

**Keywords:** Frontal cortex, Rat, Ovarian hormones, Expression analysis, Microglia activation, Postmenopausal women

## Abstract

**Background:**

The intricate interactions between the immune, endocrine and central nervous systems shape the innate immune response of the brain. We have previously shown that estradiol suppresses expression of immune genes in the frontal cortex of middle-aged ovariectomized rats, but not in young ones reflecting elevated expression of these genes in middle-aged, ovarian hormone deficient animals. Here, we explored the impact of menopause on the microglia phenotype capitalizing on the differential expression of macrophage-associated genes in quiescent and activated microglia.

**Methods:**

We selected twenty-three genes encoding phagocytic and recognition receptors expressed primarily in microglia, and eleven proinflammatory genes and followed their expression in the rat frontal cortex by real-time PCR. We used young, middle-aged and middle-aged ovariectomized rats to reveal age- and ovariectomy-related alterations. We analyzed the expression of the same set of genes in the postcentral and superior frontal gyrus of pre- and postmenopausal women using raw microarray data from our previous study.

**Results:**

Ovariectomy caused up-regulation of four classic microglia reactivity marker genes including Cd11b, Cd18, Cd45 and Cd86. The change was reversible since estradiol attenuated transcriptional activation of the four marker genes. Expression of genes encoding phagocytic and toll-like receptors such as Cd11b, Cd18, C3, Cd32, Msr2 and Tlr4 increased, whereas scavenger receptor Cd36 decreased following ovariectomy. Ovarian hormone deprivation altered the expression of major components of estrogen and neuronal inhibitory signaling which are involved in the control of microglia reactivity. Strikingly similar changes took place in the postcentral and superior frontal gyrus of postmenopausal women.

**Conclusions:**

Based on the overlapping results of rat and human studies we propose that the microglia phenotype shifts from the resting toward the reactive state which can be characterized by up-regulation of CD11b, CD14, CD18, CD45, CD74, CD86, TLR4, down-regulation of CD36 and unchanged CD40 expression. As a result of this shift, microglial cells have lower threshold for subsequent activation in the forebrain of postmenopausal women.

## Background

The intricate interactions between the immune, endocrine and central nervous systems shape the immune response within the brain [1-3]. The ovarian hormone 17β-estradiol (E2) exerts potent immunomodulatory effects in neuroinflammatory models [3-7]. Both neurons [8] and glial cells [9] express the two classical estrogen receptors (ER), ERα and ERβ, which mediate the neuroprotective [10,11] and anti-inflammatory [6,12] activities of E2. Progesterone and its metabolite allopregnanolone exert estrogen-modifying and immunomodulatory effects in injury [13,14] and inflammatory [15] models. We have previously shown that E2 suppresses the expression of genes associated with the innate immune system in the frontal cortex of middle-aged ovariectomized (OVX) rats [16]. E2-regulated immune genes encode MHC class I and class II (RT1-Aw2, Cd74), Fcγ receptors (Fcgr2a, Fcgr2b), and complement proteins (C3, C4b). In contrast, E2 has no effect on the expression of the same set of genes in the same region of young OVX rats [17] reflecting elevated expression of these immune genes in the frontal cortex of middle-aged, ovarian hormone-deficient rats. This notion is supported by the results of human [18-20] and rodent [21,22] microarray studies demonstrating up-regulation of immune genes in the cerebral cortex during the course of normal aging. Up-regulation of MHC class I and class II, toll-like receptor, complement and cytokine genes has been shown to be a characteristic feature of aging in both sexes, with proportionally higher expression in women indicating sexually dimorphic changes [20].

In this study, we explored the impact of menopause on the expression of genes related to the innate immune system in the rat and human cerebral cortex. We focused on the potential alteration of the microglia phenotype as microglial cells play pivotal roles in the initiation and regulation of the immune response. It is important to note that in the adult brain, there is no exchange of microglial cells under physiological conditions [23-25]. We took advantage of the differential expression of macrophage-associated genes in resting and activated microglia [25-28]. Although the microglial response is signal-specific, activated microglia unfold strong macrophage characteristics and express elevated levels of phagocytic [29], scavenger [30] and toll-like [31] receptors (Tlrs), MHC antigens [32]. Previous studies have established that in the case of macrophage-associated genes, mRNA expression correlates well with protein expression [27,33]. Therefore, we selected thirty-four genes including twenty-three macrophage-associated and eleven complement and cytokine genes, and analyzed their mRNA expression by real-time PCR. We found up-regulation of several macrophage-associated and some complement genes in the frontal cortex of middle-aged OVX rats. To demonstrate the relevance of these observations to human menopause, we analyzed the expression of the same set of genes using raw microarray data from the postcentral gyrus and superior frontal gyrus of pre- and postmenopausal women [20]. Data analysis revealed changes highly similar to the ones we observed in the rat menopausal model. Based on these results we characterized the microglia phenotype in the forebrain of postmenopausal women.

## Methods

### Reagents

E2 was purchased from Sigma (St. Louis, MO, USA). Alzet osmotic minipumps (model 2004) were obtained from Durect (Cupertino, CA, USA). Microfluidic cards, PCR and reverse transcription reagents were ordered from Applied Biosystems (Foster City, CA, USA).

### Experimental animals and treatments

Female Harlan-Wistar rats were purchased from Toxicoop (Budapest, Hungary). Animals were housed individually on a 12-h light/12-h dark cycle, with unrestricted access to phytoestrogen-free rodent diet (Harlan Teklad Global Diets, Madison, WI, USA). We applied four rat models: young adult, 2 month-old rats with low E2 levels (Y group), middle-aged, 13-month old intact female rats (M group), middle-aged OVX rats (M/OVX group), and middle-aged OVX rats with chronic E2 treatment (M/OVX+E2 group). For the young adult group we chose young OVX rats, since ovariectomy did not result in changes of macrophage-associated genes, as we reported earlier addressing the effect of E2 treatment in this model [17]. Bilateral ovariectomy of young (n = 10) and middle-aged (n = 20) rats was performed under deep anesthesia. Animals in the M group (n = 9) were sham-operated. After surgery, rats were housed individually and ten days later, received treatments with vehicle. E2 replacement in middle-aged OVX rats was carried out as described earlier [16].

On the day of sample preparation, animals were deeply anesthetized and perfused transcardially with 100 ml of cold fixative solution containing 10% RNA*later* in phosphate-buffered saline. In all experiments, the same procedure was followed for the preparation of the frontal cortex as published earlier [17]. Protocols were reviewed and approved by the Animal Welfare Committee of the Institute of Experimental Medicine (Number A5769-01, permission from the Department of Epidemiology and Animal Welfare, Municipal Agriculture Office, Budapest, Hungary). Experiments were carried out in accordance with the legal requirements of the European Community (Decree 86/609/EEC).

### Total RNA isolation from the frontal cortex

Total RNA was isolated from cortical samples using the RNeasy Lipid Tissue Mini Kit (Qiagen, Hilden, Germany). RNA analytics included A260nm/A280nm readings using a Nanodrop Spectrophotometer and capillary electrophoresis using RNA Nano Chips with the 2100 Bioanalyzer (Agilent, Santa Clara, CA, USA). All RNA samples displayed RNA integrity numbers above 8.2.

### Quantitative real-time PCR

Custom TaqMan low density arrays (TLDA) were designed to study in depth the regulation of thirty-four macrophage-associated and immune genes by quantitative real-time PCR. Microfluidic cards (Applied Biosystems, Santa Clara, CA, USA) were preloaded by the manufacturer with selected inventoried assays for the genes of our interest (Table 1) and for five potential house-keeping genes including 18S rRNA, glyceraldehyde-3-phosphate dehydrogenase (Gapdh), glucuronidase beta (Gusb), hypoxanthine guanine phosphoribosyl-transferase (Hprt) and peptidyl-prolyl isomerase A (Ppia). Each assay consisted of a FAM dye-labeled TaqMan MGB probe and two PCR primers. Reverse transcription and real-time PCR were run as described earlier [16]. The RealTime StatMiner (Integromics, Granada, Spain) software and relative quantification against calibrator samples (ΔΔCt) were used for analysis. To find the most stable endogenous controls, the normfinder stability scoring method [34] was used. A computed internal control corresponding to the geometric mean of cycle threshold (Ct) values of selected house-keeping genes was used for ΔCt calculation [35].

**Table 1:** Age- and ovarian hormone-related changes in expression of genes related to microglial reactivity in the frontal cortex of middle-aged female rats

**Symbol**	**Taqman assay ID**	**RQ (age)**	***P *(age)**	**RQ (OVX)**	***P *(OVX)**	**RQ (age+OVX)**	***P *(age+OVX)**
**Macrophage-associated genes**							
Phagocytic and scavenger receptors							
**Cd11b**^b^	Rn00709342_m1	**1.389**^**a**^	0.044	**2.000**^**a**^	< 0.001	**2.778**^**a**^	< 0.001
**Cd18**^b^	Rn01427948_m1	1.206		**1.488**^**a**^	0.001	**2.164**^**a**^	< 0.001
Cd93	Rn00584525_g1	1.271	0.082	0.796	0.051	1.012	
Fcgr2b	Rn00598391_m1	**1.560**^**a**^	0.024	**1.595**^**a**^	< 0.001	**2.488**^**a**^	< 0.001
Msr2	Rn01455191_m1	2.630		**1.353**^**a**^	0.025	**3.558**^**a**^	0.003
Cd36	Rn00580728_m1	**0.567**^**a**^	<0.001	**0.776**^**a**^	0.012	**0.440**^**a**^	< 0.001
Rage	Rn00584249_m1	1.225		0.870		1.066	
Recognition receptors							
Cd14	Rn00572656_g1	0.844		1.391		1.174	
**Cd40**^b^	Rn01423590_m1	1.496	0.087	0.990		1.481	0.054
**Cd45**^b^	Rn00709901_m1	1.196		**1.504**^**a**^	< 0.001	**1.799**^**a**^	< 0.001
**Cd86**^b^	Rn00571654_m1	1.178		**1.513**^**a**^	0.001	**1.782**^**a**^	< 0.001
Tlr2	Rn02133647_s1	1.015		1.000		1.015	
Tlr4	Rn00569848_m1	1.207		**1.253**^**a**^	0.006	**1.512**^**a**^	< 0.001
Tlr9	Rn01640054_m1	**1.452**^**a**^	0.033	1.348		**1.907**^**a**^	0.008
RT1-Aw2	Rn03034964_u1	**14.696**^**a**^	< 0.001	**1.479**^**a**^	0.001	**21.735**^**a**^	< 0.001
RT1-N1	Rn00561858_m1	**0.588**^**a**^	0.046	**3.300**^**a**^	< 0.001	**1.941**^**a**^	< 0.001
**Cd74**	Rn00565062_m1	**2.379**^**a**^	0.031	0.948		**2.255**^**a**^	0.047
Signaling							
Nos2	Rn00561646_m1	0.971		1.072		1.041	
Irf7	Rn01450778_g1	1.855		**2.519**^**a**^	< 0.001	**4.748**^**a**^	< 0.001
Irf9	Rn01489163_m1	**1.332**^**a**^	0.030	**1.311**^**a**^	0.004	**1.746**^**a**^	< 0.001
**Regulatory genes for microglia reactivity**							
Microglial receptors							
Cd47	Rn00569914_m1	0.884	0.084	**1.189**^**a**^	0.041	1.051	
Cd200r	Rn00576646_m1	**0.821**^**a**^	0.005	0.880	0.069	**0.722**^**a**^	< 0.001
Cx3cr1	Rn00591798_m1	**1.375**^**a**^	0.006	0.975		**1.341**^**a**^	0.005
Neuronal ligands							
Cd200	Rn00580478_m1	0.945		0.878	0.088	**0.829**^**a**^	0.019
Sirpa	Rn00564609_m1	0.922		1.071		0.987	
Cx3cl1	Rn00593186_m1	0.928		**0.813**^**a**^	< 0.001	**0.754**^**a**^	< 0.001
**Complement and cytokine genes**							
Complement							
C1qb	Rn00570480_m1	**1.739**^**a**^	< 0.001	0.951		**1.654**^**a**^	< 0.001
C1-Inh	Rn01485600_m1	1.008		**1.541**^**a**^	0.005	**1.553**^**a**^	0.009
C3	Rn00566466_m1	**4.297**^**a**^	< 0.001	**1.193**^**a**^	0.010	**5.126**^**a**^	< 0.001
Chemokine and cytokines							
Ccl2	Rn00580555_m1	1.294		**1.634**^**a**^	0.079	**2.114**^**a**^	0.014
Il1b	Rn00580432_m1	**2.390**^**a**^	< 0.001	**0.518**^**a**^	<0.001	1.238	
Il6	Rn00561420_m1	1.119		0.651		0.728	
Tgfb1	Rn00572010_m1	0.898	0.057	**1.500**^**a**^	< 0.001	**1.347**^**a**^	< 0.001
Tnf	Rn99999017_m1	0.843		1.595	0.084	1.345	

Quiescent and activated microglial cells differ strikingly in the expression of macrophage-associated genes. Age- and ovariectomy-related changes in mRNA expression were measured by real-time PCR. The applied TaqMan assays were listed. RQ represents the expression of a given gene in response to aging or ovariectomy compared to basal (control) expression. RQ (age) represents the ratio of mRNA expression in middle-aged versus young rats. RQ (OVX) shows the ratio of mRNA levels in middle-aged OVX rats versus middle-aged ones. RQ (age+OVX) values correspond to the ratio of mRNA expression in middle-aged OVX rats compared to young ones. Statistical significance of the alterations was tested by analysis of variance (ANOVA) with Newman-Keuls post hoc test, and was considered significant at *P* < 0.05; ^a^RQ values with statistically significant changes. *P*-values are indicated if < 0.1. ^b^Microglial marker genes.

### Analysis of human microarray data

Files [GEO: GSE11882] [20], contained microarray data from four regions of the forebrain: postcentral gyrus (PG), superior frontal gyrus (SG), entorhinal cortex, hippocampus. As menopause occurs between 50 and 53 years of age [36,37], this analysis included female cases divided into two age groups. The first group consisted of the putatively premenopausal subjects between 25 and 50 years of age, the second included postmenopausal women between 60 and 78 years of age. Importantly, we excluded subjects above 80 years of age since at this time significant age-related alterations occur in the cortical transcriptome. From the data set, we analyzed PG and SG, which were relevant to compare with the rat frontal cortex. Sample size (n), average age (age) in years and standard deviation of age (SD) were used to characterize the premenopausal PG (n_PG_ = 7, age_PG_ = 38.9, SD_PG_ = 7.9) and SG (n_SG_ = 10, age_SG_ = 39.1, SD_SG_ = 7.6), and postmenopausal PG (n_PG_ = 5, age_PG_ = 71.2, SD_PG_ = 4.4) and SG (n_SG_ = 6, age_SG_ = 71.7, SD_SG_ = 4.1) data. Raw microarray data were pre-processed for analysis by GC robust multi-array average (GCRMA) [38]. From the expression set, probesets were selected based on the relevance to rat data. After annotation, we identified twenty-nine human genes with high confidence (Table 2). Difference analysis of gene expression was performed by linear models combined with empirical Bayesian methods [39]; *P* was adjusted by the false discovery rate-based method [40]. In all statistical and data mining work, Bioconductor packages [41] in R-environment were used.

**Table 2:** Data analysis showed alterations in the expression of macrophage-associated, regulatory and proinflammatory genes in cortical regions of postmenopausal women, indicating overlapping changes with the rat results

		**Microarray analysis**				**Real-time PCR**	
**Symbol**	**Probeset**	**FC (PG)**	P * (P G)	**FC (SG)**	P * (S G)	**RQ (age+OVX)**	***P *(age+OVX)**
**Macrophage-associated genes**							
Phagocytic and scavenger receptors							
**CD11b**^**b**^	205786_s_at	1.341	0.918	1.145	0.832	**2.778**	< 0.001
**CD18**^**b**^	1555349_a_at	**2.808**^**a**^	0.697	**2.331**^**a**^	0.629	**2.164**	< 0.001
FCGR2b	210889_s_at	1.020	1.000	1.228	0.746	**2.488**	< 0.001
CD36	228766_at	**0.670**^**a**^	0.942	0.812	0.793	**0.440**	< 0.001
RAGE	217046_s_at	1.000	1.000	0.991	0.960	1.066	
Recognition receptors							
CD14	201743_at	**5.134**^**a**^	0.697	**2.817**^**a**^	0.629	1.174	
**CD40**^**b**^	205153_s_at	1.025	0.877	0.967	0.881	1.481	0.054
**CD45**^**b**^	212588_at	**1.903**^**a**^	0.918	1.152	0.938	**1.799**	< 0.001
**CD86**^**b**^	205685_at	1.466	0.697	1.094	0.709	**1.782**	< 0.001
TLR2	204924_at	**1.832**^**a**^	0.781	1.035	1.000	1.015	
TLR4	221060_s_at	1.152	1.000	**1.634**^**a**^	0.629	**1.512**	< 0.001
TLR9	223903_at			1.182	0.629	**1.907**	0.008
**CD74**^**b**^	1567628_at	**2.232**^**a**^	0.697	**1.631**^**a**^	0.689	**2.255**	0.047
Signaling							
NOS2	212531_at	0.965	1.000	1.002	1.000	1.041	
IRF7	208436_s_at	1.047	1.000	1.341	0.629	**4.748**	< 0.001
IRF9	203882_at	1.105	1.000	1.242	0.629	**1.746**	< 0.001
**Regulatory genes for microglia reactivity**							
Microglial receptors							
CD200R	1552875_a_at	0.990	0.942	0.990	0.763	**0.722**	< 0.001
CX3CR1	1568934_at	**0.557**^**a**^	0.967	0.960	1.000	**1.341**	0.005
Neuronal ligand							
CD200	209582_s_at	0.886	1.000	**0.497**^**a**^	0.629	**0.829**	0.019
SIRPA	202897_at	0.799	0.877	0.908	0.750	0.987	
CX3CL1	823_at	**0.462**^**a**^	0.697	**0.575**^**a**^	0.629	**0.754**	< 0.001
**Complement and cytokine genes**							
Complement							
C1q	202953_at	**4.364**^**a**^	0.697	**2.319**^**a**^	0.629	**1.654**	< 0.001
C1-INH	200986_at	1.419	0.877	**1.723**^**a**^	0.629	**1.553**	0.009
C3	217767_at	**1.577**^**a**^	0.942	1.246	0.842	**5.126**	< 0.001
Chemokine and cytokines							
CCL2	216598_s_at	**1.976**^**a**^	0.942	**2.492**^**a**^	0.629	**2.114**	0.014
IL1b	205067_at	0.669	0.984	0.743	0.763	1.238	
IL6	205207_at	**0.594**^**a**^	0.907	0.748	0.709	0.728	
TGFb1	203085_s_at	**1.812**^**a**^	0.781	**1.988**^**a**^	0.629	**1.347**	< 0.001
TNF	207113_s_at					1.345	

Differential expression of selected genes in the postcentral and superior frontal gyrus of pre- and postmenopausal women. Fold change (FC) corresponds to the ratio of the older and younger group mean expressions for a given gene. ^a^FC > 1.5; P* false discovery rate-adjusted raw *P*-value; RQ(age+OVX) values correspond to the ratio of mRNA expression in middle-aged OVX rats compared to young ones; ^b^conventional microglial marker genes are in bold.

## Results

### Age and ovarian hormone deficiency led to elevation in mRNA expression of Cd11b, Cd18, Cd45, Cd74 and Cd86 in the frontal cortex of middle-aged female rats

**Figure 1:**
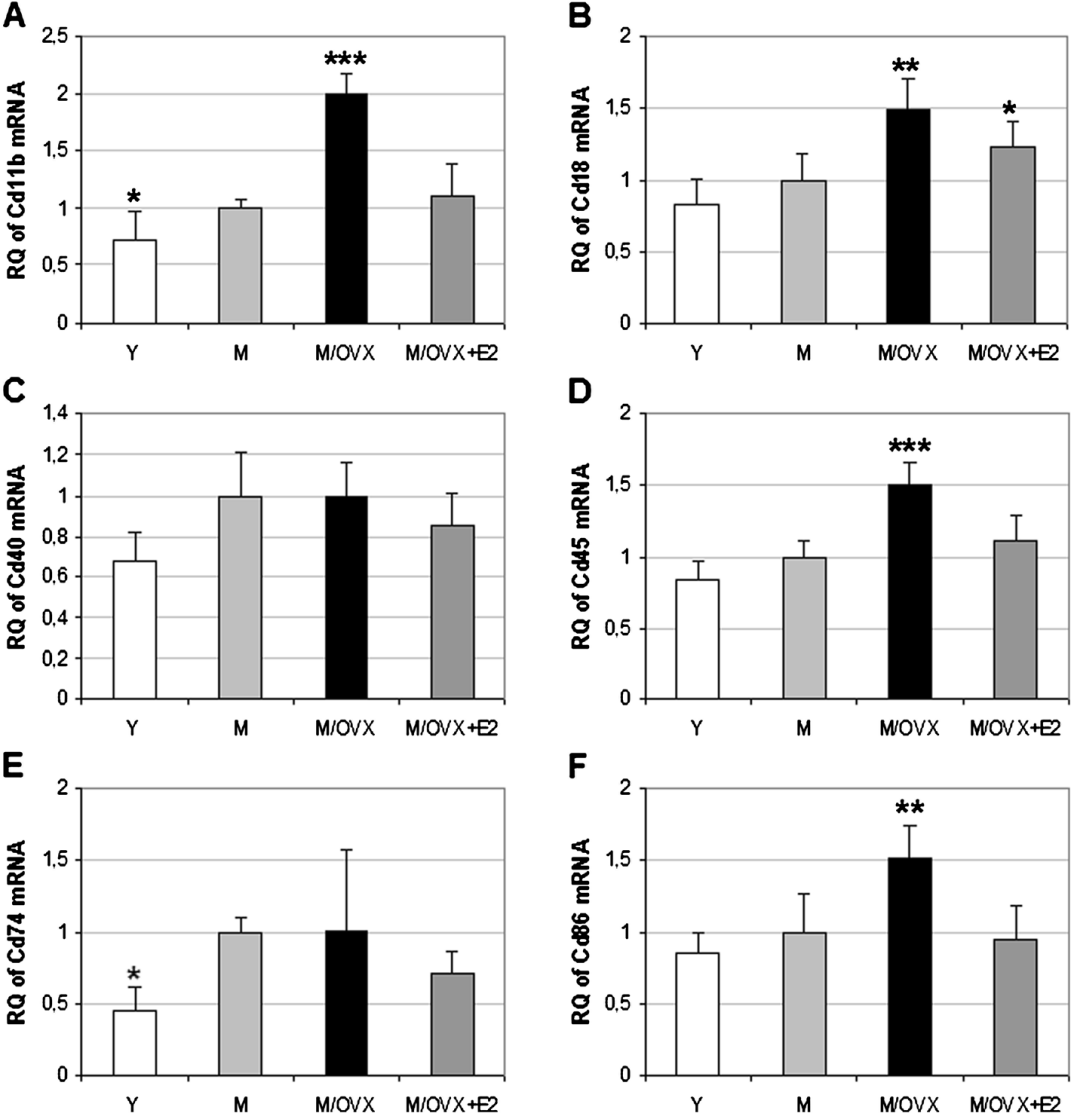
**Age- and hormone-dependent alteration in the mRNA expression of widely used microglia marker genes in the frontal cortex of middle-aged female rats.** Real-time PCR demonstrated age- and ovarian hormone-related increase in the transcription of Cd11b (**A**), Cd18 (**B**), Cd45 (**D**) and Cd86 (**F**). 17β-estradiol (E2) replacement attenuated these changes. Cd40 (**C**) and Cd74 (**E**) expression did not change after ovariectomy. Error bars show SD of six samples for each group. Statistical significance of the alterations in different groups compared to middle-aged female rats (M) was analyzed by analysis of variance (ANOVA) with Newman-Keuls post hoc test, and considered significant at *P* < 0.05. Asterisks indicate significant changes: *corresponds to 0.01<*P* <0.05, ** to 0.001<*P* <0.01 and *** to *P* < 0.001. Y, young rat; M/OVX, middle-aged ovariectomized (OVX) rat; M/OVX+E2, middle-aged OVX rat treated chronically with E2.

We explored the impact of ovarian hormone deficiency on mRNA expression of six genes encoding the alpha and beta chain of complement receptor (CR) type 3 (Cd11b and Cd18), TNF receptor superfamily member 5 (Cd40), leukocyte common antigen (Cd45), MHC class II-associated invariant chain (Cd74) and B72 antigen (Cd86). These receptors are widely considered as markers of microglia reactivity [25,27]. As a result of ovariectomy, we found a 1.5- to 2.0-fold increase in mRNA expression of Cd11b (Figure 1A), Cd18 (Figure 1B), Cd45 (Figure 1D) and Cd86 (Figure 1F). Other markers such as Cd40 (Figure 1C) and Cd74 (Figure 1E) did not change.

We determined age-related changes in the expression of these marker genes by comparing middle-aged rats to young ones. As a result of aging, we found a 1.4-fold enhancement in Cd11b (Figure 1A), 2.0-fold increase in Cd74 (Figure 1E) and 1.2-fold elevation in the other four genes. In the case of Cd11b, Cd18, Cd45 and Cd86, ovarian hormone deficiency-related alterations exceeded age-related ones underscoring the importance of ovarian hormones on microglial gene expression. Altogether, age and ovarian hormone deficiency resulted in an average of 2.0-fold elevation in the expression of the marker genes, with the exception of Cd40 (Table 1). It is noteworthy that Nos2 was not induced (Table 1).

We studied the effect of E2 replacement on the ovariectomy-dependent increase of marker genes. E2 attenuated the enhancement of Cd11b, Cd18, Cd45 and Cd86 (Figure 1). These results indicated that alterations in the expression of microglia marker genes were reversible, at least in part, following ovariectomy.

### Ovariectomy-dependent changes in the expression of genes related to phagocytosis

**Figure 2:**
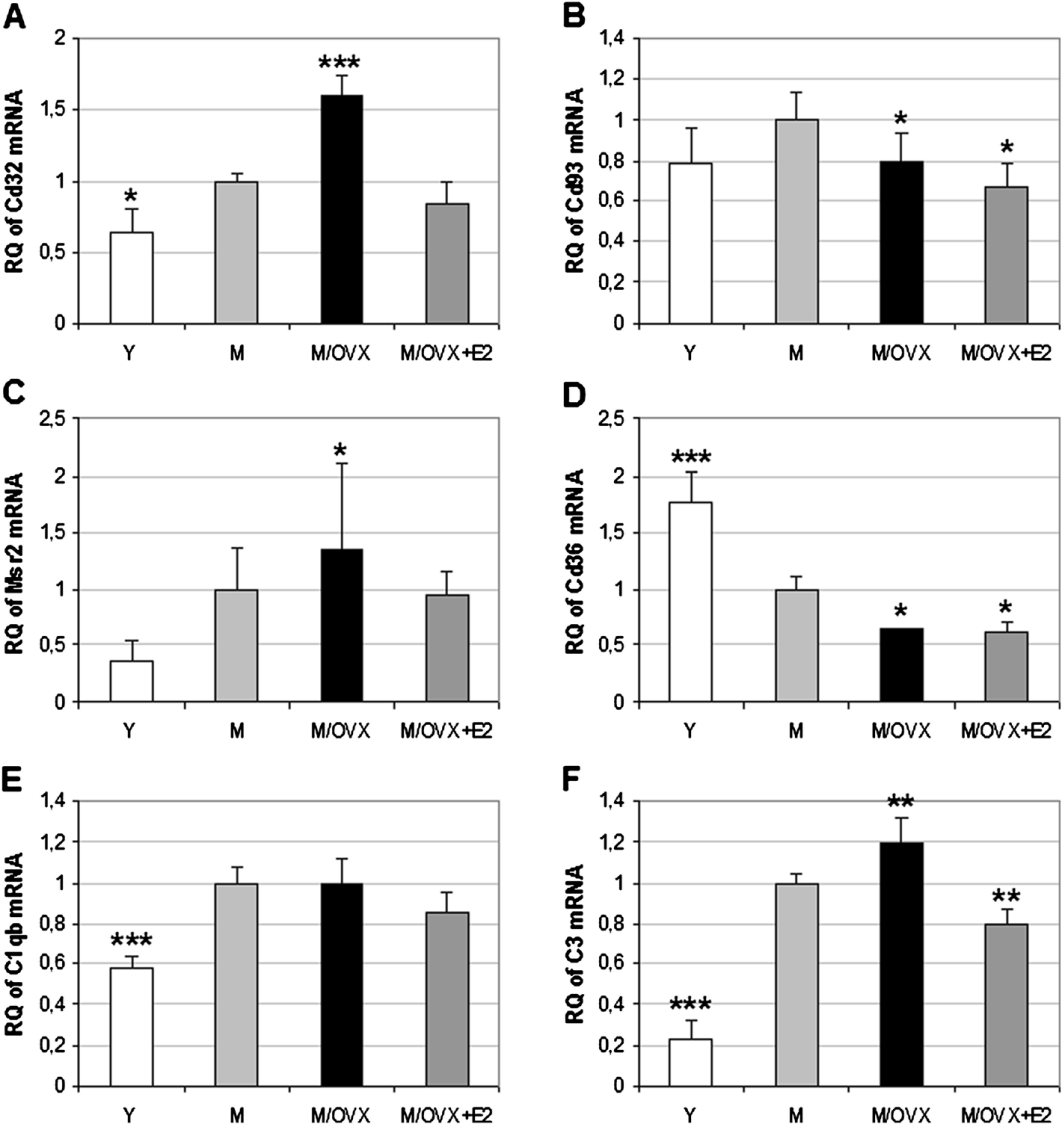
**Age- and hormone-related changes in mRNA expression of genes related to phagocytosis.** Real-time PCR demonstrated age- and ovariectomy-related increase in the expression of Cd32 (**A**), Msr2 (**C**), C1qb (**E**), C3 (**F**), and decrease of Cd93 (**B**), Cd36 (**D**). Error bars show SD of six biological samples. Statistical significance of the alterations in different groups compared to middle-aged female rats (M) was analyzed using analysis of variance (ANOVA) followed by Newman-Keuls post hoc test. Asterisks indicate changes with statistical significance: *corresponds to 0.01<*P* < 0.05, ** to 0.001<*P* < 0.01 and *** to *P* < 0.001. Y, young rat; M/OVX, middle-aged ovariectomized (OVX) rat; M/OVX+E2, middle-aged OVX rat with 17β-estradiol (E2) replacement.

In addition to Cd11b and Cd18, we examined age- and ovariectomy-related alterations in the expression of Fcγ receptor 2a (Cd32), phagocytic C1q receptor (Cd93), macrophage scavenger receptor 2 (Msr2) and leukocyte differentiation antigen (Cd36). Similar to Cd11b and Cd18, expression of Cd32 (Figure 2A), Cd93 (Figure 2B) and Msr2 (Figure 2C) showed a 1.6-, 1.3- and 2.6-fold age-related increase, respectively. Expression of Cd32 and Msr2 enhanced further, whereas Cd93 expression decreased 0.8-fold after ovariectomy. Expression of Cd36 dropped 0.6-fold in the aging brain, which decreased further following ovariectomy (Figure 2D). Altogether, age and ovarian hormone deficiency caused 2.5- and 3.6-fold increase in the expression of Cd32 and Msr2, respectively. In contrast, Cd36 decreased 0.44-fold.

Cd11b/Cd18 and Cd93 recognize C3 activation fragments and C1q, respectively. Messenger RNA expression of C1q (Figure 2E) and C3 (Figure 2F) increased in the aging frontal cortex 1.8- and 4.3-fold, respectively. The robust increase of C3 was amplified further after ovariectomy. Again, E2 reversed the increase of Cd11b, Cd18, Cd32 and Msr2 expression (Figure 2), which was in good correlation with the attenuation of Cd45 and Cd86 expression following ovariectomy (Figure 1).

### Ovarian hormone deficiency enhanced mRNA expression of toll-like receptor 4 and 9

**Figure 3:**
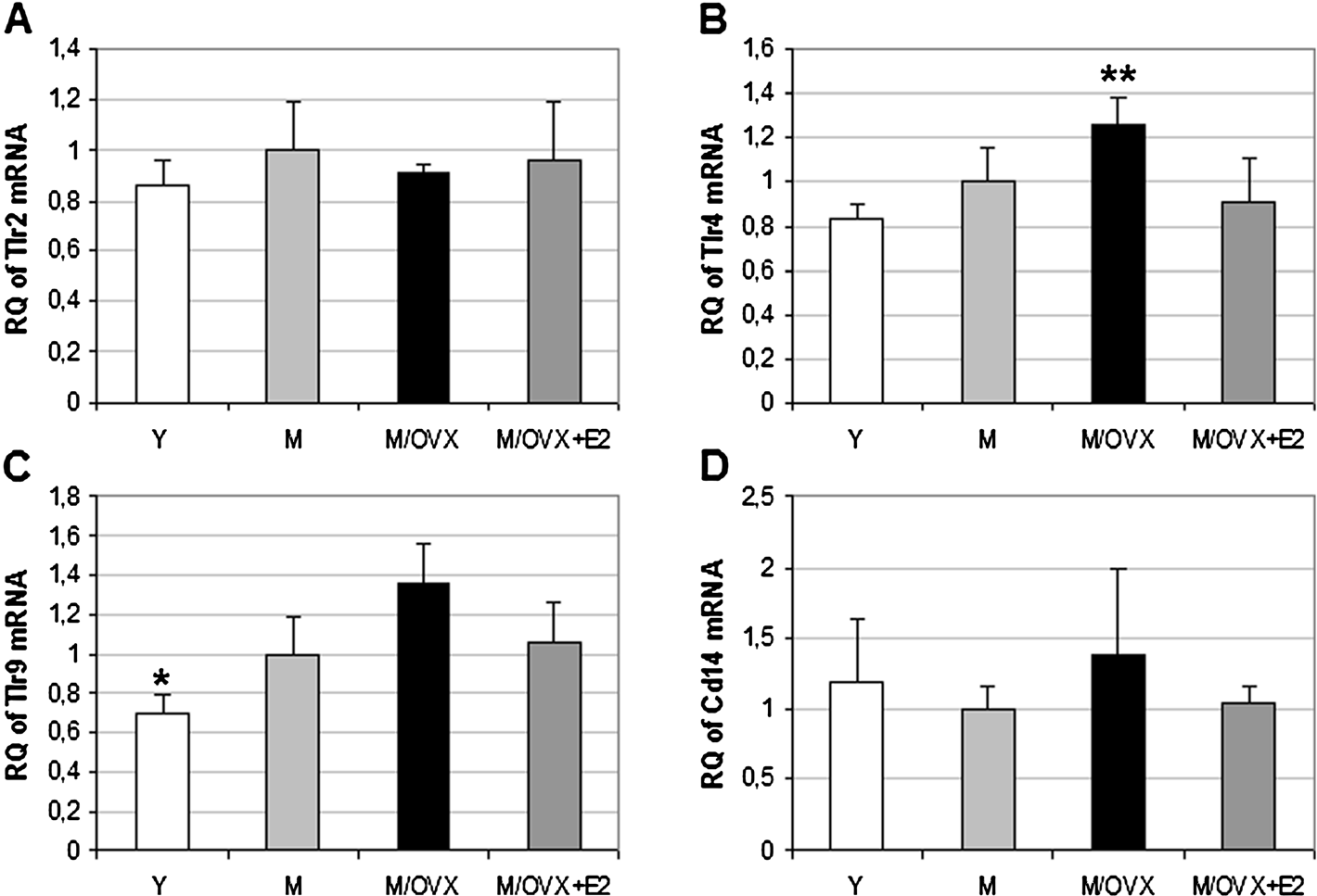
**Age- and ovarian hormone-dependent regulation of genes encoding toll-like receptors and co-receptor Cd14 in the rat frontal cortex.** TaqMan-based quantitative real-time PCR revealed changes in mRNA expression of Tlr4 (**B**) and Tlr9 (**C**). Tlr2 (**A**) and Cd14 (**D**) did not alter. Error bars show SD of six independent measurements. Statistical significance of the alterations in different groups compared to middle-aged female rats (M) was calculated using analysis of variance (ANOVA) with Newman-Keuls post hoc test, and considered significant at *P* < 0.05. Asterisks indicate changes with statistical significance: * corresponds to 0.01<*P* < 0.05, ** to 0.001<*P* < 0.01 and *** to *P* < 0.001. Y, young rat; M/OVX, middle-aged ovariectomized (OVX) rat; M/OVX+E2, middle-aged OVX rat treated with 17β-estradiol (E2).

Microglial cells express Tlrs and co-receptors to recognize apoptotic cells, amyloid peptide and bacterial cell wall components among others [25,42]. Aging led to a 1.2- to 1.5-fold increase in the expression of Tlr4 and Tlr9. Ovariectomy caused comparable elevation (Figure 3B and C). Altogether, age and ovarian hormone deficiency resulted in 1.5- and 2.0-fold increase in the expression of Tlr4 and Tlr9. Changes in the expression of Tlr2 (Figure 3A) and Cd14 (Figure 3D) did not reach statistical significance.

Again, E2 attenuated the increase of Tlr4 and Tlr9 expression similarly to Cd11b, Cd18, Cd32, Cd45, Cd86 and Msr2.

### Aging and ovarian hormone deprivation increased mRNA expression of RT1-Aw2

MHC class I antigens RT1-Aw2 and RT1-N1 showed significant age- and ovariectomy-related increase in their expression (Table 1). RT1-Aw2 showed 14.7- and 1.5-fold age- and ovariectomy-related increase, respectively. Altogether, there was a 21.7-fold increase in mRNA expression of RT1-Aw2.

### Age and ovariectomy altered the expression of genes involved in the regulation of microglia reactivity

**Figure 4:**
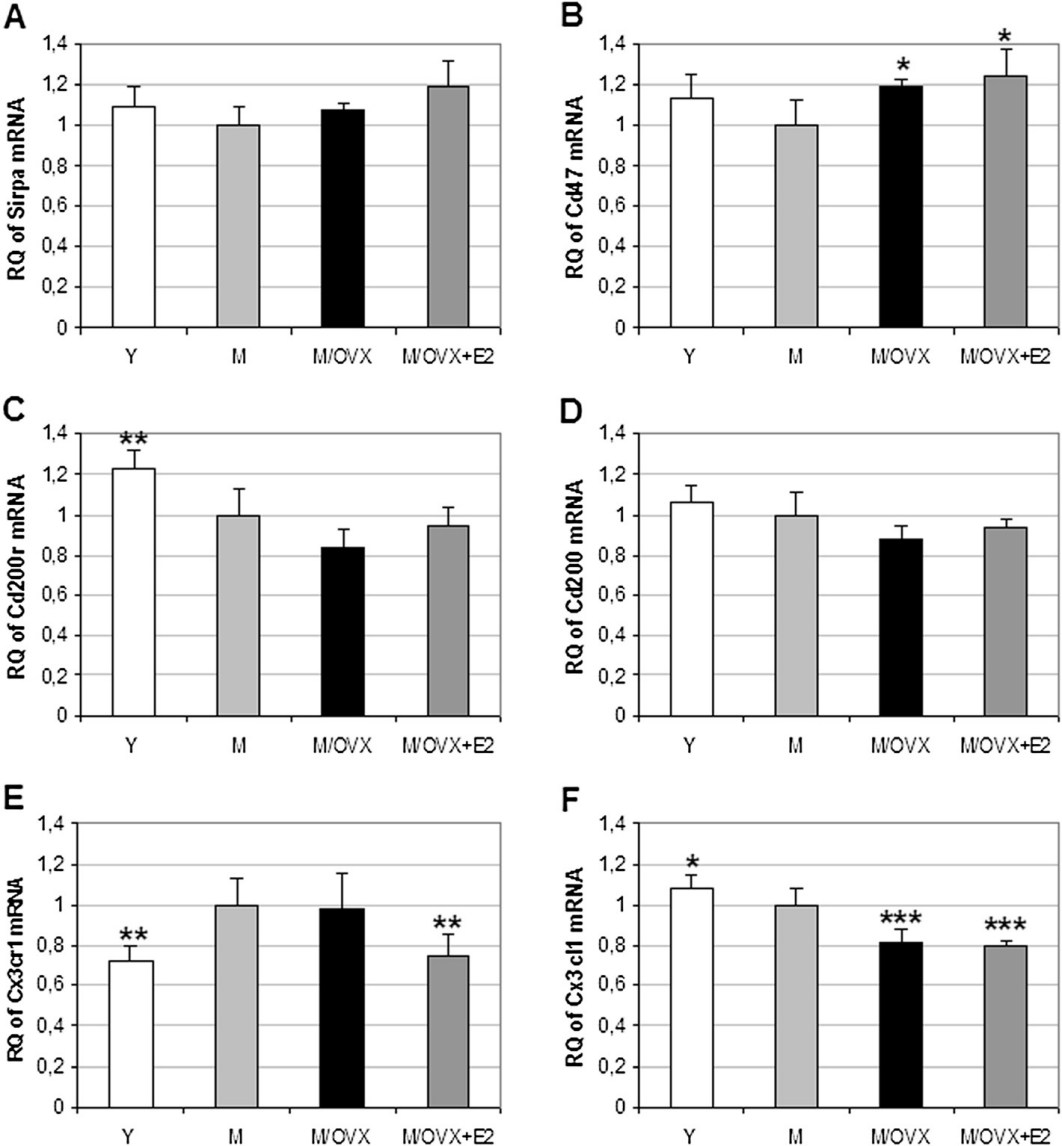
**Age- and ovarian hormone-dependent regulation of genes encoding microglial receptors and their inhibitory neuronal ligands in the rat frontal cortex.** TaqMan-based quantitative real-time PCR revealed no change in Sirpa (**A**), increase in the expression Cd47 (**B**), decrease in mRNA expression of Cd200r (**C**), Cd200 (**D**) and Cx3cl1 (**F**). Cx3cr1 (**E**) increased during aging. Error bars show SD of six independent measurements. Statistical significance of the alterations in different groups compared to middle-aged female rats (M) was determined by analysis of variance (ANOVA) with Newman-Keuls post hoc test, and considered at *P* <0.05. Asterisks indicate changes with statistical significance: *corresponds to 0.01<*P* < 0.05, ** to 0.001<*P* < 0.01 and *** to *P* < 0.001. Y, young rat; M/OVX, middle-aged OVX rat; M/OVX+E2, middle-aged ovariectomized (OVX) rat treated with 17β-estradiol (E2).

We examined age- and ovarian hormone deficiency-related alterations of key components of neuronal inhibitory pathways and estrogen signaling, which play a pivotal role in the regulation of microglia reactivity. Neurons tightly control the reactivity of microglial cells by expressing inhibitory ligands [43]. We measured expression of six genes encoding neuronal ligand-microglial receptor pairs such as Cd47-Sirpa, Cd200-Cd200r and Cx3cl1-Cx3cr1. As a result of aging and ovariectomy, we found 0.8- to 0.7-fold decrease in the expression of Cd200 (Figure 4D) and Cd200r (Figure 4C). We observed a 0.75-fold decrease in Cx3cl1 (Figure 4F), and a 1.3-fold increase in Cx3cr1 (Figure 4E). There was no change in Cd47 (Figure 4B) and Sirpa (Figure 4A).

**Figure 5:**
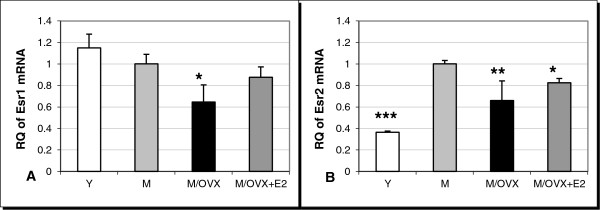
**Age- and ovarian hormone-dependent regulation of mRNA expression of estrogen receptors in the rat frontal cortex.** We measured age- and hormone-dependent changes by TaqMan-based quantitative real-time PCR in mRNA expression of Esr1 (**A**) and Esr2 (**B**), encoding ERα and ERβ, respectively. Error bars show SD of six independent measurements. Asterisks indicate changes with statistical significance: *corresponds to 0.01<*P* < 0.05, ** to 0.001<*P* < 0.01 and *** to *P* < 0.001. Y, young rat; M, middle-aged female rat; M/OVX, middle-aged ovariectomized (OVX) rat; M/OVX+E2, middle-aged OVX rat with 17β-estradiol (E2) replacement.

We also determined age- and ovarian hormone-dependent alterations in the expression of Esr1 and Esr2 genes encoding ERα and ERβ, respectively. Both age and ovarian hormone deficiency altered their expression. Expression of Esr1 decreased (Figure 5A), whereas Esr2 increased (Figure 5B) during aging. Ovariectomy decreased the transcription of both receptors. Altogether, aging and ovariectomy caused a 0.6-fold decrease in Esr1 and a 1.8-fold increase in Esr2 expression.

E2 replacement ten days after ovariectomy rescued the transcription of Esr1 and Esr2. These results suggested that key genes in control mechanisms for microglia reactivity were altered in the frontal cortex of middle-aged, ovarian hormone-deprived rats.

### Expression of selected cytokines and other immune genes

We studied the expression of three pro-inflammatory cytokines (Il1b, Il6, Tnf) and astrocytic Tgfb1. The expression of Il1b increased during aging and dropped after ovariectomy. Tgfb1 did not change during aging, but was enhanced significantly after ovariectomy. Il6 and Tnf showed no significant alteration. As a result of aging and ovariectomy, only Tgfb1 was enhanced 1.3-fold, indicating that a characteristic pro-inflammatory milieu did not develop in the ovarian hormone-deprived aging cortex.

We examined the expression of Ccl2 and found 1.6-fold ovariectomy-related elevation in its expression, which was reversed by E2 (Table 1). Irf7 and Irf9, encoding IFN regulatory factors, increased during aging and intensified further after ovariectomy (Table 1).

### Microarray data analysis revealed strikingly similar changes of gene expression in the postcentral and superior frontal gyrus of postmenopausal women

To address the impact of menopause on gene expression in the human forebrain, we carried out an analysis of raw microarray data from our previous gene expression profiling study [20]. The expression of twenty-nine genes associated with the innate immune response was compared in the PG and SG from premenopausal versus postmenopausal women with an average age of 39 and 71 years, respectively. To identify differentially expressed genes in small samples, we used the raw FC values, as FC correlates well with reproducibility [44]. We considered alterations with FC > 1.5 to be reliable changes. Our analysis revealed up-regulation of CD14, CD18, CD45, TLR2, TLR4, CD74, C1q, C3, CCL2, and down-regulation of CD36, CX3CR1, CX3CL1 and CD200 in postmenopausal women (Table 2).

## Discussion

In this study, we demonstrated that menopause amplified the age-related increase in the expression of macrophage-associated genes in the frontal cortex. From the major findings we conclude that i) the microglia phenotype shifts from the resting towards the activated state in a rat model of menopause, ii) the shift is reversible, iii) altered expression of phagocytic receptors may indicate modified phagocytic activity, iv) impairment of regulatory mechanisms may contribute to the early state of microglia activation, and v) strikingly similar changes occur in the forebrain of postmenopausal women.

### The microglia phenotype shifts from the resting toward the activated state in the frontal cortex of middle-aged ovariectomized rats

Slight up-regulation of Cd11b, Fcgr2b, Tlr9, RT1-Aw2, and Cd74 in the frontal cortex of middle-aged rats indicates an initial age-related alteration in microglial gene expression. Elevated expression of C1q, C3 and Il1b is in accord with previous studies [18-22]. As these genes are expressed in astrocytes and microglia, it is likely that changes of glial phenotypes are intertwined. It is worth noting that the expression of C1-Inh, encoding the sole regulator of the classical activation pathway [45], does not change, suggesting that the control of the classical pathway may be impaired.

Ovarian hormone deficiency enhanced the expression of Cd11b, Cd18, Cd32, Cd45, Cd86, Tlr4, RT1-Aw2, and decreased Cd36, reflecting an initial shift in the microglia phenotype. The increase in the expression of IFN regulatory factors Irf7 and Irf9 is in accord with the shift of microglia from the resting phenotype [46]. Down-regulation of Cd36 [47] is also a characteristic feature of the acquired microglia phenotype. We suggest that down-regulation of Cd36 may underlie the decreased internalization of amyloid-β by aged compared to young microglia [48].

Our human microarray data analysis identified strikingly similar changes. Based on these results we propose that in the PG and SG of postmenopausal women the microglia phenotype is characterized by the up-regulation of CD11b, CD14, CD18, CD45, CD74, CD86, TLR4, and down-regulation of CD36. Notably, the expression of CD40 and NOS2 does not change.

### The effect of E2 indicates that the shift in microglia phenotype is reversible

E2 replacement attenuated the ovarian hormone deprivation-related increase in the expression of Cd11b, Cd18, Fcgr2b, Msr2, Cd45, Cd86, Tlr4, RT1-Aw2 and RT1-N1. Down-regulation of these macrophage-associated genes suggests that E2 may attenuate microglial activation. This notion is consistent with the regulatory role of E2 on macrophage functions [49] and microglia activation in inflammatory [12,50] and injury [51] models.

### Complement-mediated phagocytosis may increase in the middle-aged cortex

Age-dependent elevation takes place in the expression of C1q, and its phagocytic receptor CD93 in the frontal cortex of female rats. C1q binds to pathogens and apoptotic cells, directly or through antibodies and pentraxins [45]. C1q binding initiates the classical pathway of complement resulting in recruitment of phagocytes, phagocytosis of apoptotic cells and destruction of invading pathogens. In the brain C1q also recognizes and binds to proteins with pathogenic conformation, such as amyloid-β and prion protein. Elevated expression of C1q may facilitate early recognition and phagocytosis of pathogenic substances in the aging brain.

C3 is the central component of the complement system [45]. Activation pathways converge at C3, and its proteolytic fragments are ligands for complement receptors on various cell types including microglia. The interaction between C3 fragment iC3b and CR3 links complement activation and phagocyte functions. In the presence of C3 activators, elevated expression of C3 and CR3 may contribute to early steps of microglia activation, often referred to as microglial priming [2]. This notion is supported by the co-localization of C3b fragments and activated microglia in humans and in rodent models of neuroinflammatory diseases [52-54]. The impact of the interaction between C3 fragments and CR3 on microglia priming has been recently demonstrated in a multiple sclerosis model [54].

### Neuronal inhibitory pathways and estrogen signaling are altered after menopause

Neuronal inhibitory ligands play a pivotal role in the tight control of microglia reactivity [24,43]. We demonstrated age- and ovariectomy-related alterations in the expression of inhibitory ligands, including down-regulation of Cx3cl1 and Cd200, and up-regulation of Cd47 in the frontal cortex of middle-aged rats. The expression of microglial receptors for these ligands also showed changes, including down-regulation of Cd200r and up-regulation of Cx3cr1. Decreased expression of Cd200r is in accord with the age-related decrease in the expression of CD200R protein in the mouse brain [55]. CD200 fusion protein decreases microglia activation in the hippocampus of aged rats [56]. These data suggest that decreased expression of Cd200 and Cd200r may contribute to the increased expression of macrophage-associated genes. In the PG and SG of postmenopausal women, down-regulation of CD200 and CX3CL1 also indicates the impairment of major regulatory mechanisms for the control of microglia reactivity.

Estrogen signaling is also involved in the regulation of microglia reactivity [4,50]. Direct regulation is supported by the presence of ERα and ERβ in microglial cells [9] and by the well-known effects of E2 on macrophages [49]. However, ERα and ERβ are also expressed in neurons, astrocytes [57], and oligodendrocytes [58], so the role of indirect effects cannot be ruled out either. Here, we provide evidence for inverse age-related regulation of ERα and ERβ. Decreased expression of ERα together with the declining levels of E2 is likely to reduce estrogen signaling in the aging rat cortex following ovariectomy. However, we found no sign of alteration of ESR1 and ESR2 expression in the forebrain of postmenopausal women.

Summing up, the results provide evidence for microglial activation in the cortex of middle-aged rats following surgical menopause. Based on the overlapping changes from rodent and human studies, we propose that in the forebrain of postmenopausal women the microglia phenotype shifts from the resting towards the reactive state, which is characterized by up-regulation of CD11b, CD18, CD45, CD74, CD86, TLR4, down-regulation of CD36 and unchanged CD40 expression. This early state of activation, called microglial priming, seems to be reversible, as E2 replacement attenuates the expression of macrophage-associated genes in the rat frontal cortex. Microglia priming results in a phenotype with a lower threshold for subsequent activation [2,59]. It is proposed that in the presence of primed microglia, systemic infection and inflammation pose a higher threat for the aging brain [59].

## Abbreviations

ANOVA: Analysis of variance; CR3: Complement receptor type 3; Cd11b: Alpha chain of CR3; Cd14: Monocyte differentiation antigen; Cd18: Beta chain of CR3; Cd32: Fcγ receptor 2a; Cd36: Leukocyte differentiation antigen; Cd40: TNF receptor superfamily member 5; Cd45: Leukocyte common antigen; Cd74: MHC class II-associated invariant chain; Cd86: B72 antigen; Cd93: Phagocytic C1q receptor; E2: 17β-estradiol; FC: Fold change; Gapdh: Glyceraldehyde-3-phosphate dehydrogenase; Gusb: Glucuronidase beta; Hprt: Hypoxanthine guanine phosphoribosyl-transferase; IFN: Interferon; Msr2: Macrophage scavenger receptor 2; OVX: Ovariectomized; PCR: Polymerase chain reaction; PG: Postcentral gyrus; Ppia: Peptidyl-prolyl isomerase A; RQ: Relative quantity; SG: Superior frontal gyrus; TLDA: TaqMan low density array; Tlr: Toll-like receptor; TNF: Tumor necrosis factor.

## Competing interests

The authors declare that they have no competing interests.

## Authors’ contributions

MS, ZL designed the study. MS, EH, and IK collected the tissues; IK isolated the frontal cortices. MS, EH, and IL ran and analyzed real-time PCR. NB, and CC collected the microarray data. NS performed microarray data analysis. MS, and ZL wrote the manuscript. All authors have read and approved the final version of the manuscript.
